# Genome sequence of the acid-tolerant *Burkholderia sp.* strain WSM2230 from Karijini National Park, Australia

**DOI:** 10.4056/sigs.5008793

**Published:** 2013-12-31

**Authors:** Robert Walker, Elizabeth Watkin, Rui Tian, Lambert Bräu, Graham O’Hara, Lynne Goodwin, James Han, Elizabeth Lobos, Marcel Huntemann, Amrita Pati, Tanja Woyke, Konstantinos Mavromatis, Victor Markowitz, Natalia Ivanova, Nikos Kyrpides, Wayne Reeve

**Affiliations:** 1 School of Biomedical Sciences, Faculty of Health Sciences, Curtin University, Western Australia, Australia; 2 Centre for Rhizobium Studies, School of Veterinary and Life Sciences, Murdoch University, Western Australia, Australia; 3 School of Life and Environmental Sciences, Deakin University, Victoria, Australia; 4 Los Alamos National Laboratory, Bioscience Division, Los Alamos, New Mexico, USA; 5 DOE Joint Genome Institute, Walnut Creek, California, USA; 6 Biological Data Management and Technology Center, Lawrence Berkeley National Laboratory, Berkeley, California, USA

**Keywords:** root-nodule bacteria, nitrogen fixation, rhizobia, *Betaproteobacteria*

## Abstract

*Burkholderia sp.* strain WSM2230 is an aerobic, motile, Gram-negative, non-spore-forming acid-tolerant rod isolated from acidic soil collected in 2001 from Karijini National Park, Western Australia, using *Kennedia coccinea* (Coral Vine) as a host. WSM2230 was initially effective in nitrogen-fixation with *K. coccinea*, but subsequently lost symbiotic competence. Here we describe the features of *Burkholderia sp.* strain WSM2230, together with genome sequence information and its annotation. The 6,309,801 bp high-quality-draft genome is arranged into 33 scaffolds of 33 contigs containing 5,590 protein-coding genes and 63 RNA-only encoding genes. The genome sequence of WSM2230 failed to identify nodulation genes and provides an explanation for the observed failure of the laboratory grown strain to nodulate. The genome of this strain is one of 100 sequenced as part of the DOE Joint Genome Institute 2010 Genomic Encyclopedia for Bacteria and *Archaea*-Root Nodule *Bacteria* (GEBA-RNB) project.

## Introduction

*Burkholderia* spp. are ubiquitous in the environment and are found in nearly all terrestrial and some marine ecosystems. They have adapted to occupy numerous niches and may have saprophytic, parasitic, pathogenic or symbiotic lifestyles [1]. Emerging evidence suggests an ancient and stable symbiosis between *Burkholderia* and *Mimosa* genera within South America [2] and between *Burkholderia* and legumes from the *Papilionoideae* subfamily in South Africa [3,4]. Despite this, there is very little data regarding the symbiosis between *Burkholderia* and endemic legumes outside of South America and South Africa.

In Australia, legumes are predominately nodulated by species from the genera *Bradyrhizobium*, *Ensifer*, and *Rhizobium* [5,6]. There are no published genomes or species descriptions of symbiotic *Burkholderia* spp. isolated in Australia and there is a paucity of information on the interaction between *Burkholderia* and endemic Australia legumes. *Burkholderia sp.* WSM2230 was isolated from an effective nitrogen fixing nodule on *Kennedia coccinea* grown in an acidic soil (pH(CaCl_2_) 4.8) collected from Karijini National Park, Western Australia. Its symbiotic phenotype was authenticated in glasshouse experiments (Watkin, unpublished). Recently this isolate was revived from long-term storage from frozen glycerol stocks but failed to form nodules on *K. coccinea* in axenic glasshouse trials (Walker, unpublished). In this regard, it is interesting that the South African microsymbiont *B. tuberum* STM678^T^ only infrequently forms effective nodules on *Macroptilium atropurpureum* (Siratro). A recent study [7] revealed that *B. tuberum* forms effective nodules on Siratro when water levels are reduced and temperature is increased. Unlike *B. tuberum* STM678^T^, the annotation of the genome sequence of the laboratory cultured strain of WSM2230 failed to identify nodulation genes and this offers an explanation for the lack of a nodulation phenotype.

Establishing the genomic sequences of Australian *Burkholderia* will be beneficial to understand the mutualistic interactions occurring between plant and rhizosphere organisms in low-pH soil. WSM2230 was only isolated from Karijini National Park acidic soil (pH(CaCl_2_) 4.8) and other sites where the soil pH was higher (pH(CaCl_2_) >7) did not contain any *Burkholderia* symbionts. In these more alkaline soils, numerous *Bradyrhizobium* and *Rhizobium* spp. were instead trapped (Watkin, unpublished). Soil pH is an edaphic variable that controls microbial biogeography [8] and the acid tolerance of *Burkholderia* has been shown to account for the biogeographical distribution of this genus [9].

The genome of WSM2230 is one of two Australian *Burkholderia* genomes (the other being that of WSM2232 (GOLD ID Gi08832)) that have now been sequenced through the Genomic Encyclopedia for *Bacteria* and *Archaea*-Root Nodule Bacteria (GEBA-RNB) program. Here we present a preliminary description of the general features of the *Burkholderia sp.* WSM2230 together with its genome sequence and annotation. The genomes of WSM2232 and WSM2230 will be an important resource to identify the processes enabling such isolates to adapt to the infertile, highly acidic soils that dominate the Australian landscape.

## Classification and features

**Figure 1:**
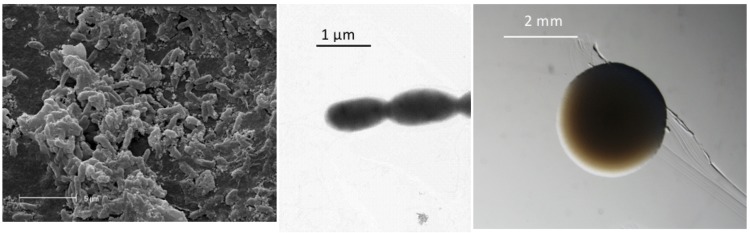
Images of *Burkholderia sp.* strain WSM2230 using scanning (Left) and transmission (Center) electron microscopy and the appearance of colony morphology on a solid medium (Right).

*Burkholderia sp.* strain WSM2230 is a motile, non-sporulating, non-encapsulated, Gram-negative rod in the order *Burkholderiales* of the class *Betaproteobacteria*. The rod-shaped form varies in size with dimensions of 0.5 μm for width and 1.0-2.0 μm for length (Figure 1 Left and Center). It is fast growing, forming colonies within 1-2 days when grown on LB agar [10] devoid of NaCl and within 2-3 days when grown on half strength Lupin Agar (½LA) [11], tryptone-yeast extract agar (TY) [12] or a modified yeast-mannitol agar (YMA) [13] at 28°C. Colonies on ½LA are -opaque, slightly domed and moderately mucoid with smooth margins (Figure 1 Right).

**Figure 2:**
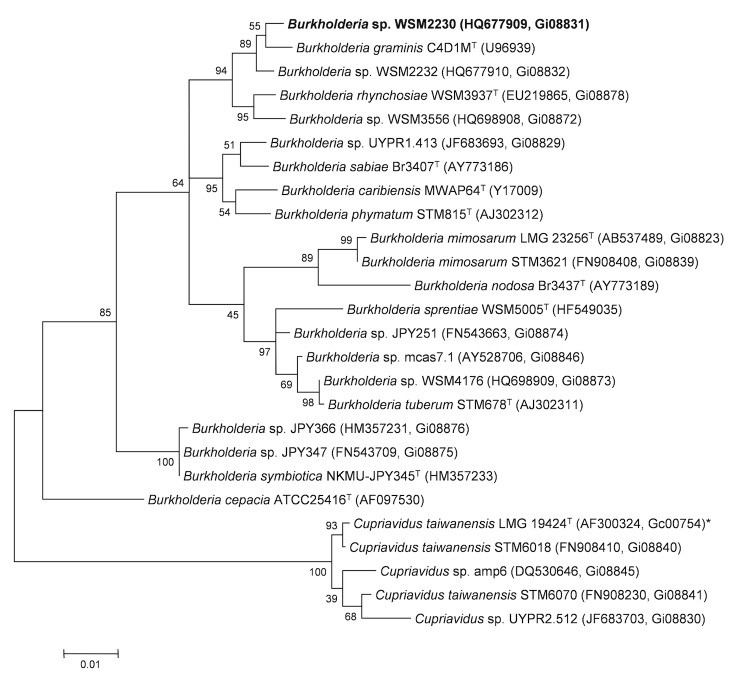
Phylogenetic tree showing the relationship of *Burkholderia sp.* strain WSM2230 (shown in bold print) to other members of the order *Burkholderiales* based on aligned sequences of the 16S rRNA gene (1,242 bp internal region). All sites were informative and there were no gap-containing sites. Phylogenetic analyses were performed using MEGA [25], version 5. The tree was built using the Maximum-Likelihood method with the General Time Reversible model [26]. Bootstrap analysis [27] with 500 replicates was performed to assess the support of the clusters. Type strains are indicated with a superscript T. Brackets after the strain name contain a DNA database accession number and/or a GOLD ID (beginning with the prefix G) for a sequencing project registered in GOLD [28]. Published genomes are indicated with an asterisk.

*Burkholderia sp.* WSM2230 can solubilize inorganic phosphate, produces hydroxymate-like siderophores, and can tolerate a pH range of 4.5 - 9.0 (Walker, unpublished). Minimum Information about the Genome Sequence (MIGS) is provided in Table 1. Figure 2 shows the phylogenetic neighborhood of *Burkholderia sp.* strain WSM2230 in a 16S rRNA sequence based tree. This strain shares 99% (1352/1364 bp) sequence identity to the 16S rRNA gene of the sequenced strain *Burkholderia sp.* WSM2232 (Gi08831).

**Table 1:** Classification and general features of *Burkholderia sp.* strain WSM2230 according to the MIGS recommendations [14]

**MIGS ID**	**Property**	**Term**	**Evidence code**
	Current classification	Domain *Bacteria*	TAS [15]
		Phylum *Proteobacteria*	TAS [16]
		Class *Betaproteobacteria*	TAS [17,18]
		Order *Burkholderiales*	TAS [18,19]
		Family *Burkholderiaceae*	TAS [18,20]
		Genus *Burkholderia*	TAS [21-23]
		Species *Burkholderia sp.*	IDA
		Strain WSM2230	IDA
	Gram stain	Negative	IDA
	Cell shape	Rod	IDA
	Motility	Motile	IDA
	Sporulation	Non-sporulating	NAS
	Temperature range	Mesophile	IDA
	Optimum temperature	30°C	IDA
	Salinity	Non-halophile	IDA
MIGS-22	Oxygen requirement	Aerobic	IDA
	Carbon source	Varied	IDA
	Energy source	Chemoorganotroph	NAS
MIGS-6	Habitat	Soil, root nodule, on host	IDA
MIGS-15	Biotic relationship	Free living, symbiotic	IDA
MIGS-14	Pathogenicity	Non-pathogenic	IDA
	Biosafety level	1	IDA
	Isolation	Root nodule of *Kennedia coccinea*	IDA
MIGS-4	Geographic location	Karijini National Park, Australia	IDA
MIGS-5	Soil collection date	September 2001	IDA
MIGS-4.1 MIGS-4.2	Latitude Longitude	117.99 -22.5	IDA IDA
MIGS-4.3	Depth	0-10 cm	IDA
MIGS-4.4	Altitude	Not reported	

Evidence codes – IDA: Inferred from Direct Assay; TAS: Traceable Author Statement (i.e., a direct report exists in the literature); NAS: Non-traceable Author Statement (i.e., not directly observed for the living, isolated sample, but based on a generally accepted property for the species, or anecdotal evidence). These evidence codes are from the Gene Ontology project [24].

### Symbiotaxonomy

*Burkholderia sp.* WSM2230 formed nodules (Nod+) on, and fixed N_2_ (Fix+) with, *K. coccinea* when first isolated. However, after long term storage and its subsequent culture, it failed to nodulate Australian legume hosts (Table 2).

**Table 2:** Compatibility of WSM2230 with nine legume species for nodulation (Nod) and N_2_-Fixation (Fix)

**Species name**	**Common name**	**Growth type**	**Nod**	**Fix**	**Reference**
*K. coccinea*	Coral Vine	Perennial	+^1^	+^1^	IDA
*Swainsona formosa*	Sturts Desert Pea	Annual	-	-	IDA
*Indigofera trita*	-	Annual	-	-	IDA
*Acacia acuminata*	Jam Wattle	Perennial	-	-	IDA
*A. paraneura*	Weeping Mulga	Perennial	-	-	IDA

^1^result obtained from trapping experiment but the isolate failed to nodulate after long term storage.

IDA: Inferred from Direct Assay from the Gene Ontology project [24].

## Genome sequencing and annotation

### Genome project history

This organism was selected for sequencing on the basis of its environmental and agricultural relevance to issues in global carbon cycling, alternative energy production, and biogeochemical importance, and is part of the Community Sequencing Program at the U.S. Department of Energy, Joint Genome Institute (JGI) for projects of relevance to agency missions. The genome project is deposited in the Genomes OnLine Database [28] and an improved-high-quality-draft genome sequence in IMG. Sequencing, finishing and annotation were performed by the JGI. A summary of the project information is shown in Table 3.

**Table 3:** Genome sequencing project information for *Burkholderia sp.* WSM2230

**MIGS ID**	**Property**	**Term**
MIGS-31	Finishing quality	Improved high-quality draft
MIGS-28	Libraries used	1x Illumina library
MIGS-29	Sequencing platforms	Illumina HiSeq 2000
MIGS-31.2	Sequencing coverage	Illumina: 368×
MIGS-30	Assemblers	Velvet version 1.1.04; Allpaths-LG version r39750
MIGS-32	Gene calling methods	Prodigal 1.4
	GOLD ID	Gi08831
	NCBI project ID	165309
	Database: IMG	2513237151
	Project relevance	Symbiotic N_2_ fixation, agriculture

### Growth conditions and DNA isolation

*Burkholderia sp.* strain WSM2230 was cultured to mid logarithmic phase in 60 ml of TY rich medium on a gyratory shaker at 28°C [29]. DNA was isolated from the cells using a CTAB (Cetyl trimethyl ammonium bromide) bacterial genomic DNA isolation method [30].

### Genome sequencing and assembly

The genome of *Burkholderia sp.* strain WSM2230 was sequenced at the Joint Genome Institute (JGI) using Illumina technology [31]. An Illumina standard shotgun library was constructed and sequenced using the Illumina HiSeq 2000 platform which generated 15,498,652 reads totaling 2,324 Mbp.

All general aspects of library construction and sequencing performed at the JGI can be found at the JGI user home [30]. All raw Illumina sequence data was passed through DUK, a filtering program developed at JGI, which removes known Illumina sequencing and library preparation artifacts (Mingkun, L., Copeland, A. and Han, J., unpublished). The following steps were then performed for assembly: (1) filtered Illumina reads were assembled using Velvet [32] (version 1.1.04), (2) 1–3 Kbp simulated paired end reads were created from Velvet contigs using wgsim (https://github.com/lh3/wgsim), (3) Illumina reads were assembled with simulated read pairs using Allpaths–LG [33] (version r39750). Parameters for assembly steps were: 1) Velvet --v --s 51 --e 71 --i 2 --t 1 --f "-shortPaired -fastq $FASTQ" --o "-ins_length 250 -min_contig_lgth 500"), 2) wgsim (-e 0 -1 76 -2 76 -r 0 -R 0 -X 0), 3) Allpaths–LG (STD_1,project,assembly,fragment,1,200,35,,,inward,0,0 SIMREADS,project,assembly,jumping,1,,,3000,300,inward,0,0).

The final draft assembly contained 33 contigs in 33 scaffolds. The total size of the genome is 6.3 Mbp and the final assembly is based on 2,324 Mbp of Illumina data, which provides an average 368× coverage of the genome.

### Genome annotation

Genes were identified using Prodigal [34] as part of the DOE-JGI annotation pipeline [35], followed by a round of manual curation using the JGI GenePrimp pipeline [36]. The predicted CDSs were translated and used to search the National Center for Biotechnology Information (NCBI) non-redundant database, UniProt, TIGRFam, Pfam, PRIAM, KEGG, COG, and InterPro databases. The tRNAScanSE tool [37] was used to find tRNA genes, whereas ribosomal RNA genes were found by searches against models of the ribosomal RNA genes built from SILVA [38]. Other non–coding RNAs such as the RNA components of the protein secretion complex and the RNase P were identified by searching the genome for the corresponding Rfam profiles using INFERNAL [39]. Additional gene prediction analysis and manual functional annotation was performed within the Integrated Microbial Genomes (IMG-ER) platform [40,41].

## Genome properties

The genome is 6,309,801 nucleotides 63.07% GC content (Table 4) and comprised of 33 scaffolds (Figures 3a,3b,3c and Figure 3d) of 33 contigs. From a total of 5,653 genes, 5,590 were protein encoding and 63 RNA only encoding genes. The majority of genes (83.42%) were assigned a putative function whilst the remaining genes were annotated as hypothetical. The distribution of genes into COGs functional categories is presented in Table 5.

**Table 4:** Genome Statistics for *Burkholderia sp.* strain WSM2230

**Attribute**	**Value**	**% of Total**
Genome size (bp)	6,309,801	100.00
DNA coding region (bp)	5,480,804	86.86
DNA G+C content (bp)	3,979,790	63.07
Number of scaffolds	33	
Number of contigs	33	
Total gene	5,653	100.00
RNA genes	63	1.11
rRNA operons*	1	0.02
Protein-coding genes	5,590	98.89
Genes with function prediction	4,716	83.42
Genes assigned to COGs	4,614	81.62
Genes assigned Pfam domains	4,843	85.67
Genes with signal peptides	571	10.10
Genes with transmembrane helices	1,343	23.76
CRISPR repeats	0	

*4 copies of 5S, 2 copies of 16S and 1 copy of 23S rRNA.

**Figure 3a:**
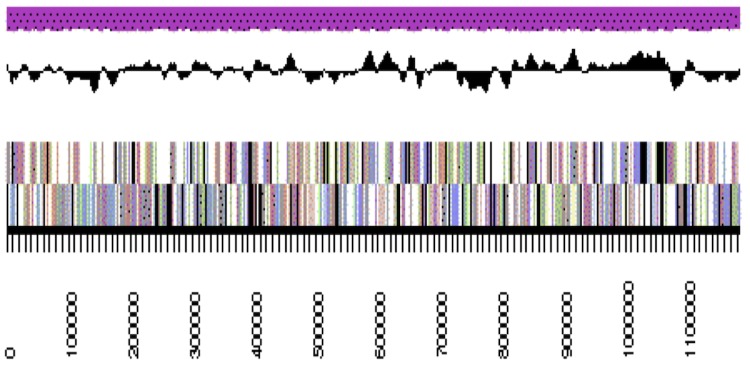
Graphical map of WSM2230_A3ACDRAFT_scaffold_0.1 of the genome of *Burkholderia sp.* strain WSM2230. From bottom to the top of each scaffold: Genes on forward strand (color by COG categories as denoted by the IMG platform), Genes on reverse strand (color by COG categories), RNA genes (tRNAs green, sRNAs red, other RNAs black), GC content, GC skew.

**Figure 3b:**
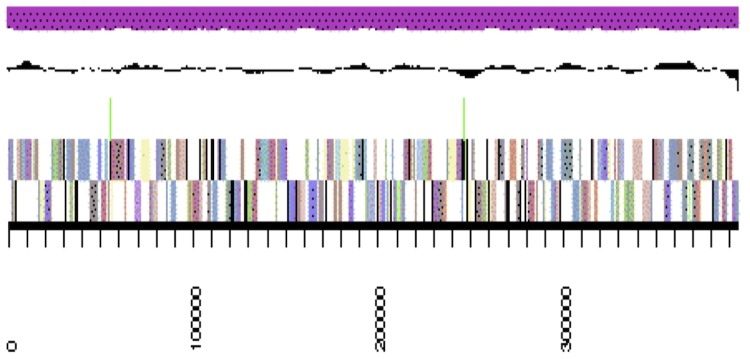
Graphical map of WSM2230_A3ACDRAFT_scaffold__3.4 of the genome of *Burkholderia sp.* strain WSM2230. From bottom to the top of each scaffold: Genes on forward strand (color by COG categories as denoted by the IMG platform), Genes on reverse strand (color by COG categories), RNA genes (tRNAs green, sRNAs red, other RNAs black), GC content, GC skew.

**Figure 3c:**
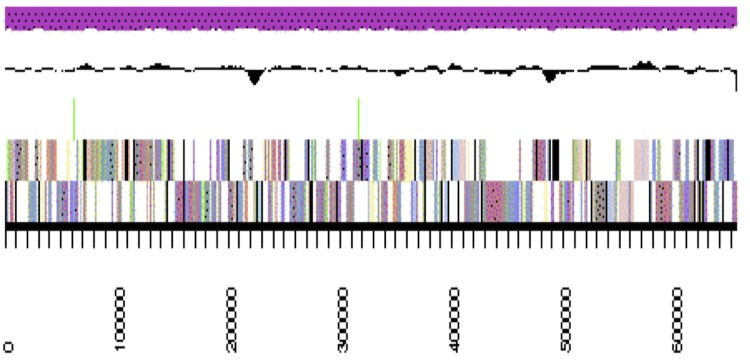
Graphical map of WSM2230_A3ACDRAFT_scaffold_1.2 of the genome of *Burkholderia sp.* strain WSM2230. From bottom to the top of each scaffold: Genes on forward strand (color by COG categories as denoted by the IMG platform), Genes on reverse strand (color by COG categories), RNA genes (tRNAs green, sRNAs red, other RNAs black), GC content, GC skew.

**Figure 3d:**
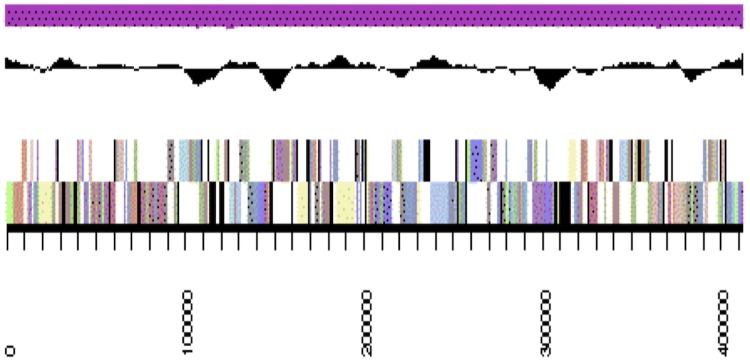
Graphical map of WSM2230_A3ACDRAFT_scaffold_2.3 of the genome of *Burkholderia sp.* strain WSM2230. From bottom to the top of each scaffold: Genes on forward strand (color by COG categories as denoted by the IMG platform), Genes on reverse strand (color by COG categories), RNA genes (tRNAs green, sRNAs red, other RNAs black), GC content, GC skew.

**Table 5:** Number of protein coding genes of *Burkholderia sp.* strain WSM2230 associated with the general COG functional categories

**Code**	**Value**	**%age**	**Description**
J	179	3.46	Translation, ribosomal structure and biogenesis
A	2	0.04	RNA processing and modification
K	474	9.17	Transcription
L	141	2.73	Replication, recombination and repair
B	1	0.02	Chromatin structure and dynamics
D	40	0.77	Cell cycle control, cell division, chromosome partitioning
Y	0	0.0	Nuclear structure
V	47	0.91	Defense mechanisms
T	260	5.03	Signal transduction mechanisms
M	357	6.90	Cell wall/membrane/envelope biogenesis
N	103	1.99	Cell motility
Z	0	0.00	Cytoskeleton
W	2	0.04	Extracellular structures
U	128	2.48	Intracellular trafficking, secretion, and vesicular transport
O	169	3.27	Posttranslational modification, protein turnover, chaperones
C	371	7.17	Energy production and conversion
G	395	7.64	Carbohydrate transport and metabolism
E	496	9.59	Amino acid transport and metabolism
F	95	1.84	Nucleotide transport and metabolism
H	197	3.81	Coenzyme transport and metabolism
I	271	5.24	Lipid transport and metabolism
P	233	4.51	Inorganic ion transport and metabolism
Q	173	3.35	Secondary metabolite biosynthesis, transport and catabolism
R	610	11.80	General function prediction only
S	427	8.26	Function unknown
-	1,039	18.38	Not in COGs
